# The role of the communicated treatment rationale on treatment outcome: study protocol for a randomized controlled trial

**DOI:** 10.1186/s13063-023-07557-w

**Published:** 2023-08-17

**Authors:** Liv Henrich, Marcel Wilhelm, Philipp Lange, Winfried Rief

**Affiliations:** Department of Psychology, Division of Clinical Psychology and Psychotherapy, Philipps University of Marburg, Gutenbergstraße 18, 35032 Marburg, Germany

**Keywords:** Depression, Treatment expectations, Pharmacological placebo, Psychological placebo, Treatment rationale, Adults

## Abstract

**Background:**

Placebo effects are a well-established phenomenon in the treatment of depression. However, the mechanism underlying these effects are not fully understood. Treatment expectations are considered one explanation for why placebos work. Treatment expectations are likely to be affected by clinician-patient interactions. This study aims to investigate the role of the communicated treatment rationale in modulating treatment expectations and its effects on the treatment outcomes of a pharmacological and a psychological active placebo intervention for depression. In this study, treatment expectations are modulated by presenting illness models that are either congruent or incongruent with the treatment intervention that follows.

**Methods:**

This 2 × 2 randomized controlled trial will involve patients with major depression. Participants will either receive a biological or a psychological illness model from a clinician. Following this, they are randomly assigned to receive either a pharmacological or a psychological active placebo intervention. The illness model and the treatment are either congruent or incongruent with each other, resulting in four groups. In addition, a natural course control group will be included.

**Discussion:**

This study will provide insights into the mechanism of expectation modulation in active placebo treatments for major depression. The results may provide insights for clinicians to improve their communication with patients by focusing on treatment expectations. By identifying the factors that contribute to placebo effects, this study has the potential to improve the effectiveness of existing depression treatments and reduce the burden of this highly prevalent mental health condition.

**Trial registration:**

This trial has been registered prospectively at ClinicalTrials.gov under the identifier: NCT04719663. Registered on January 22, 2021.

**Supplementary Information:**

The online version contains supplementary material available at 10.1186/s13063-023-07557-w.

## Administrative information

Note: the numbers in curly brackets in this protocol refer to SPIRIT checklist item numbers. The order of the items has been modified to group similar items (see http://www.equator-network.org/reporting-guidelines/spirit-2013-statement-defining-standard-protocol-items-for-clinical-trials/).

**Table:** 

Title {1}	The role of the communicated treatment rationale on treatment outcome in patients with major depression: study protocol for a randomized controlled trial.
Trial registration {2a and 2b}.	ClinicalTrials.gov Identifier: NCT04719663, registered on January, 22, 2021
Protocol version {3}	NCT04719663, Version 1, registered January, 22, 2021
Funding {4}	Funded by the German Research Foundation (Deutsche Forschungsgemeinschaft; DFG) – Project-ID 422744262-TRR 289. Study materials and wages of the research and the clinical staff are paid by the funding body.
Author details {5a}	All authors: Department of Psychology, Division of Clinical Psychology and Psychotherapy, Philipps University of Marburg, Gutenbergstraße 18, 35032 Marburg, Germany Correspondence: liv.henrich@uni-marburg.de
Name and contact information for the trial sponsor {5b}	Department of Psychology, Division of Clinical Psychology and Psychotherapy, Philipps University of Marburg, Gutenbergstraße 18, 35,032 Marburg, Germany
Role of sponsor {5c}	The sponsor and funding body have no role in study design, data collection, analysis, interpretation or in writing of publications.

## Introduction

### Background and rationale {6a}

Placebo control groups in clinical trials on depression yield impressive improvements [1–5]. The difference between antidepressant drug arms and placebo arms is often small [6], especially when drug arms are compared to active placebo arms [7]. Depending on study design and methodology, it is estimated that placebo effects account for up to 82% of the antidepressants’ effectiveness [3–5, 8]. Yet, there has been little experimental research on the underlying mechanisms explaining this effect.

Treatment expectations as the main mechanism driving placebo effects could explain the lack of differences between placebo and treatment groups [5, 9–12]. Particularly active placebo groups are effective in inducing positive treatment expectations and thus placebo effects. A likely explanation is that active placebos cause minor side effects, which lead patients to believe that they have received the actual treatment drug [13].

Another explanation could be that placebo effects in pharmacological trials are merely a result of natural course of the disorder, i.e., spontaneous remission [14–16], or statistical artifacts like regression to the mean [17]. Unfortunately, few drug trials include a natural course control group, which makes it difficult to evaluate this explanation [16]. One exception is a study by Leuchter and colleagues (2014), which included a minimal care (natural course) arm besides an active antidepressant arm and a placebo arm. The results showed that both the drug and the placebo arm performed substantially better than the natural course arm [18]. This may lend some evidence to the suggestion that improvements in placebo arms are not merely attributable to spontaneous remission, but to placebo mechanisms, such as expectation effects.

Major depressive disorder (MDD) is among the most common psychological disorders [19]. Globally, the burden caused by depressive disorders has been further exacerbated by the COVID-19 pandemic, especially affecting women and younger people [20]. At a personal level, it is linked to severe impairments in terms of psychological well-being and social functioning [21]. These estimations highlight the importance of improving the uptake and the efficacy of evidence-based treatments for depression.

Both antidepressant drugs and psychological interventions present evidence-based treatments for depression that render moderate short-term, pre-post effect sizes [2, 22]. Unfortunately, anti-depressant medication is often accompanied by unwanted and potentially serious side effects [23, 24], while psychotherapy is often preceded by long waiting times [25] and requires a greater time and motivational commitment.

Experimental research on the affective system highlights the critical role of expectations in modulating emotional responses to sadness in the treatment of depression. For instance, Haas and colleagues (2020) manipulated expectations using a placebo nasal spray in individuals with major depression, which led to a reduction in emotional response to a sadness induction task [26]. Similarly, Rebstock and colleagues (2020) showed that the nasal spray placebo intervention could reduce rumination and experienced sadness in healthy individuals [27]. Both studies suggest that expectations significantly impact emotional responses to sadness, underscoring the importance of considering expectations in the treatment of depression.

Expectations regarding psychotherapy also render an important and direct effect on treatment success, even beyond the effect of therapeutic alliance [28]. Thus, psychological interventions with very different treatment foci notoriously show similar treatment efficacies [29]. It has even been suggested that common factors rather than specific treatment factors are the major agents of change in successful therapies [30, 31].

Taken together, both in pharmacological and in psychological trials, specific treatment factors are difficult to disentangle from common treatment factors, such as expectation effects. Modifying treatment expectations, therefore, presents an opportunity not only for studying the mechanisms that may underlie placebo responses but also for boosting treatment outcomes in patients with depression.

Expectations can be modified via several pathways: personal experience, observational learning, and verbal instruction/information, e.g., during clinician-patient interactions [10, 32, 33]. A common verbal instruction that occurs prior to treatment (e.g., a course of antidepressants or a therapy intervention) is the clinician-administered provision of a treatment rationale. In those encounters, a model for the etiology and the maintaining factors of the disorder are typically provided. Lebowitz and Appelbaum (2019) showed that clinicians tend to have an “either or” attitude along the biological vs psychological causal explanation dimension [34]. Ahn and colleagues (2009) showed that the stronger clinicians believed in a biological cause, the more they expected medication rather than psychotherapy to be an effective treatment [35].

Patients’ causal attributions of the disorder also contribute to treatment expectations. For instance, biomedical causal attributions for psychological disorders have been associated with prognostic pessimism and higher doubts about treatability [36, 37]. A recent psychotherapy seems to support psychological etiological beliefs, while a family history of psychological disorders seems to predict biological causal beliefs [38]. Although many patients would agree with multi-causal explanations for depressive disorders [39], anxious and depressed patients tend to have a preference for psychological treatments [40], which may not always agree with their primary care provider’s beliefs about the etiology and best course of action or the information they are given during consultations.

Only few longitudinal studies have investigated the possible associations between illness beliefs, treatment preferences, treatment expectations, and actual treatment outcomes. Experimentally, Lebowitz and Ahn (2014) could show that clinicians who were presented with vignettes of patients with mental disorders and either a biomedical or a psychosocial explanation rated the effectiveness for psychotherapy lower, and the effectiveness of medication higher, when they were presented with the biomedical explanation [41]. Similarly, Lüllmann and colleagues (2011) showed that when psychosis patients were presented with a biological causal model for schizophrenia, they showed higher willingness to take medication. Patients presented with a psychological model on the other hand reported higher personal control over symptoms [42].

To summarize, better understanding clinician-patient interactions is essential for several reasons: clinicians’ beliefs might impact the choice of treatment and how convincingly a treatment rationale is conveyed to the patient. From the patient’s perspective, these interactions are important, because they may have the power to alter critical treatment-related factors, such as treatment expectations and adherence and may ultimately determine the success of the treatment. Patients will likely have pre-existing illness beliefs about their symptoms which affect expectations about their treatability. However, the experimental research to date also suggests that these expectations are modifiable and that it is worthwhile to do so in order to boost treatment outcomes.

Thus, if expectation effects can boost or deplete the prospect of a successful treatment for depression, it is necessary to both establish a better understanding of the underlying mechanisms of this effect and test how to optimize treatment expectations during the clinician-patient encounters that are already routinely taking place.

### Objectives {7}

Broadly, this study aims to examine the mechanism of expectation modulation and its effect on outcome in active placebo treatments (pharmacological and psychological) for major depression. More specifically, the aim of this trial is to investigate whether treatment expectations affect the outcome of pharmacological and psychological interventions in depression and whether this expectation is modulated by the therapist-delivered illness model and treatment rationale. This will be done in a 2 × 2 design where patients receive either a biological (monoamine hypothesis) or a psychological (deficits in emotion regulation) illness model in the consultation with a clinician, which is then followed either by a pharmacological (Buscopan©, Butylscopolamine 10 mg) or a psychological (emotional writing) active placebo intervention. These simplified illness rationales are used because they reflect the kind of information that patients receive during the typical short interactions in general care and on the internet. The study design is furthermore extended by a natural course control group in order to be able to compare the effects of the placebo interventions to natural changes in the disorder.

Specifically, this study aims to examine (1) whether expectations for (placebo) treatments for depression can be modified by presenting illness explanations that are either congruent or incongruent with the suggested treatment rationale and (2) whether modified treatment expectations affect treatment outcomes after a 4-week active placebo intervention.

### Hypotheses

1. Providing a treatment-congruent illness rationale leads to a better outcome than providing treatment-incongruent rationales.
2. Psychological causal explanations of depression lead to a better outcome than biological explanations if a psychological intervention is provided. Biological causal explanations of depression lead to a better outcome than psychological explanations if medication is provided as treatment.
3. The treatment rationale provided by the clinician modifies patients’ treatment outcome expectations. Biological rationales increase the expectation for the outcome of pharmacological treatments, while psychological rationales increase positive outcome expectations for psychological interventions.
4. Treatment-congruent explanations reduce the risk of side effect development, in particular in the pharmacological treatment arm.
5. Interindividual differences in the effect of the provided treatment rationale are associated with pre-treatment experiences and expectations, depression severity, comorbid anxiety, and psychobiological markers (i.e., cortisol, alpha-amylase).

### Trial design {8}

In this participant and rater-blinded randomized controlled trial (RCT), four parallel experimental treatment arms are compared to each other. Equal sample sizes are allocated to all groups (*N* = 30). Superiority of the four experimental conditions over the natural course group (5th group) is assumed as well as superiority of the two congruent treatment arms over the incongruent treatment arms.

**Fig. 1:**
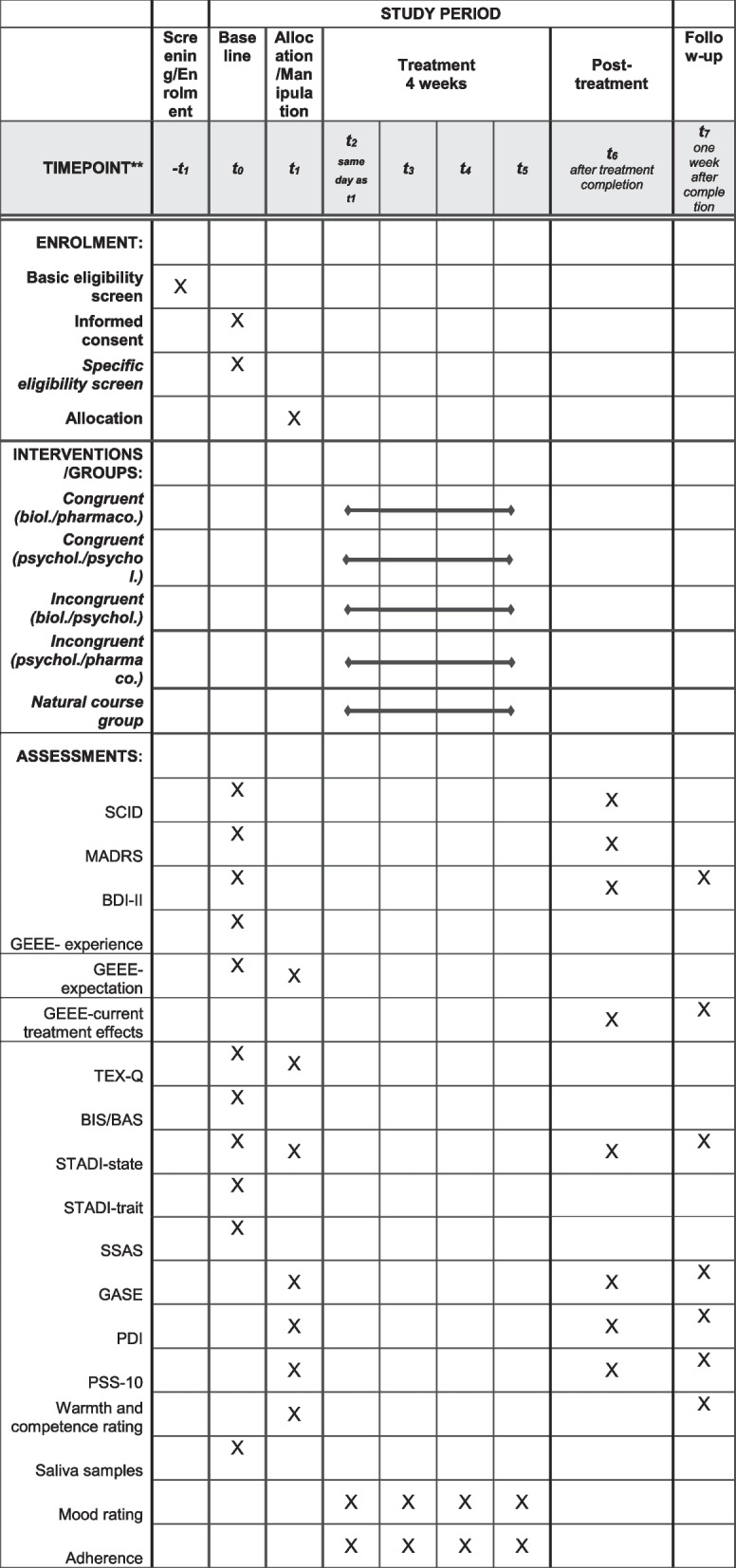
Schedule of enrolment, interventions, and assessments (SPIRIT figure). MADRS, Montgomery Asberg Depression Scale; BDI-II, Beck Depression Inventory-II; GEEE, generic rating scale for previous treatment experiences, treatment expectations, and treatment effects; TEX-Q, Treatment Expectation Questionnaire; BIS/BAS, Behavioral Inhibition/Behavioral Approach System; STADI, State-Trait-Anxiety-Depression-Inventory; SSAS, Somatosensory Amplification Scale; GASE, Generic Assessment of Side-Effects; PDI, Pain Disability Index; PSS-10, Perceived Stress Scale-10

This protocol is reported according to the standard protocol items: recommendation for interventional trials (SPIRIT) guidelines. The SPIRIT figure detailing the schedule of enrollment, interventions, and assessments is provided in Fig. 1.

## Methods: participants, interventions, and outcomes

### Study setting {9}

The study takes place at the Philipps University of Marburg (Germany) department of Clinical Psychology and Psychotherapy.

### Eligibility criteria {10}

In order to render the findings of this trial applicable to clinical practice, eligibility criteria are chosen to reflect typical clinical populations routinely treated for major depression in outpatient treatment centers. Individuals with a diagnosis of major depression (according to the SCID for DSM-5 diagnostic interview criteria), aged 18 to 80, who are fluent in German, and give their informed consent, are eligible to participate. Comorbidity is allowed, as long as major depression is the dominant clinical feature. Concordant medication is also allowed (with the exception of benzodiazepines), as long as the medication is not contraindicated with Buscopan and kept constant for at least 4 weeks preceding the trial and for the duration of the trial.

Exclusion criteria are comprised of the following: patients with severe depression (indicated by BDI-II scores and the SCID-diagnostic screening), any indications of acute suicidality, psychosis (current or lifetime), significant neurological diseases, other mental or physical disorders with substantial influence on disability, benzodiazepine intake, any intolerance against Buscopan, and sucrose or medical condition/treatment conflicting with Buscopan intake. The medical examination is performed by medical doctors, the diagnostic interviews are conducted by psychologists (psychotherapists in training), and interventions are provided by psychologists and psychology students (research staff).

### Who will take informed consent? {26a}

Written informed consent will be collected by the study physicians at the first study visit, before the medical examination and the psychological diagnostics take place. If informed consent is not given, the participant will not proceed to the screening procedures.

### Additional consent provisions for collection and use of participant data and biological specimens {26b}

Cortisol and alpha amylase samples are also collected during the course of this trial. These samples will be analyzed as part of an overarching project that aggregates data from several sub-projects in this collaborative research center (CRC). Consent for obtaining cortisol and alpha amylase samples is requested separately after diagnostic screening. Participants who do not give written consent for these samples are still allowed to partake in the current trial.

## Interventions

### Explanation for the choice of comparators {6b}

One aim of this study is to assess the size of the placebo effect in the treatment of depression more generally. Since major depression is routinely treated with pharmacological agents (antidepressants) and/or with psychotherapy, two active placebo treatments are compared to each other: an active pharmacological placebo and an active psychological placebo.

#### Active pharmacological placebo choice

We aim to analyze the role of medical pre-information about the disorder on subsequent treatments independently of the selected drug. Therefore, the active placebo pill should not have direct effects on the brain. This is the case for Buscopan (Butylscopolamine 10 mg), a non-prescription drug, that induces small side effects that resemble those of antidepressants (slightly increased risk of “dry mouth”), but that does not cross the blood–brain barrier. No serious side effects are known. Treatment duration is 4 weeks with one pill per day.

#### Active psychological placebo choice

“Emotional Writing” consists of writing about highly emotional experiences, especially those never disclosed to others (four 30-min sessions; one session per week). It is based on the theory that the suppression of emotions leads to psychological distress, originally proposed by Pennebaker [43]. According to the theory, expressive writing about deeply emotional memories should decrease distress. To date, the effectiveness of this method for treating depressive symptoms is questioned. Several meta-analytic studies on expressive writing for dealing with stressful or traumatic events report low or non-significant effect sizes for emotional writing [44–47]. A meta-analysis including 39 RCTs did not find significant long-term effects for reducing depressive symptoms either [48]. Additionally, a study on placebo effects of expressive writing showed that the technique only rendered beneficial effects if a convincing rationale was provided, indicating that the therapeutic effects of the technique do not extend beyond the placebo effect [49]. Therefore, expressive writing was chosen as a suitable psychological placebo intervention for this trial.

#### Natural course group

Few clinical studies in the field of antidepressant placebo research include a natural course control group and thus are not able to differentiate between placebo effects and spontaneous remission in the course of the disorder [10]. To control for potential regression to the mean and spontaneous improvement in the outcome variables, we employ a natural course group.

The main aim of this study is to assess how a placebo response in the treatment of depression can be modified by manipulating the patient-clinician communication during the communicated illness and treatment rationale. Thus, the main comparators are the intervention groups who receive congruent vs. incongruent illness and treatment rationales.

### Intervention description {11a}

The 2 × 2 (plus 1) design (biological/psychological treatment rationale × pharmacological/psychological treatment) for the intervention groups renders four intervention arms:

1. Congruent illness rationale and treatment: biological/pharmacological
2. Incongruent illness rationale and treatment: psychological/pharmacological
3. Congruent illness rationale and treatment: psychological/psychological
4. Incongruent illness rationale and treatment: biological/pharmacological

Upon randomization, one of the following illness rationales is provided to the participant.

### Illness rationales

#### Biological illness rationale

Depression is described as a brain disorder, and the role of monoamine dysfunction and structural and functional brain abnormalities are depicted as central mechanisms of relevance for its etiology and treatment. Biological processes are illustrated using typical charts and visualizations. Psychological influences are mentioned, but only as a byproduct of the disorder.

#### Psychological illness rationale

Depression is described as a psychological disorder resulting from emotion regulation deficits. The suppression of emotions is emphasized as a central mechanism for the development and maintenance of depression. These psychological processes are illustrated using charts and visualizations. Biological aspects are mentioned but only as a byproduct of the disorder.

Participants are provided with a handout containing the main information and illustrations of their respective illness rationales. Furthermore, they are asked to briefly summarize the rationale in their own words and highlight information that was new or especially relevant to them. This serves as a manipulation check. They further have the opportunity to ask questions about the illness rationale.

Following the provision of the illness rationale, participants receive one of the following treatment rationales and the first round of treatment.

### Treatment (active placebo) rationales

#### Pharmacological treatment

The rationale for this treatment is briefly explained as “stimulating a biological balance in people with depression, using a well-tolerated drug similar to Buscopan, which is well-known in pain treatments.” Furthermore, information on possible side effects (mouth dryness) and instructions on how to take the medication (once daily, at the same time of day) and how to deal with adherence lapses are provided. Participants are encouraged to set a reminder for the pill-intake on their phone.

Subsequently, a week’s supply of Buscopan is handed out. Once a week during the intervention period, participants pick up the next week’s supply of pills. In each of these short encounters, the research staff ask about any changes in mood or potential suicidality. Yet, the interaction is kept as short as possible, in order to avoid cross-over effects to the psychological placebo condition.

#### Active psychological placebo treatment

The rationale for this treatment is briefly explained as “improving the ability to deal with emotions to achieve a psychological balance in humans with depression.” Afterwards, the first emotional writing session follows and specific instructions based on Pennebaker’s original study for emotional writing are given (write for 30 min; grammar is not important; a range of topics is suggested).

For the emotional writing session, the study instructor presents standard psychotherapeutic attitudes (warm and encouraging demeanor; available to answer questions about the emotional writing) but will not read the written work. For the duration of the intervention period, participants complete one emotional writing session per week. The study instructor also asks about changes in mood and potential suicidality at each writing session (an exact transcript of the illness and treatment rationales can be provided upon request).

### Criteria for discontinuing or modifying allocated interventions {11b}

Participants can withdraw their written consent at any point and discontinue the study. Other reasons for discontinuation are as follows: acute suicidality and/or referral to a psychiatric hospital, a drastic increase in depressive symptoms, and adverse reactions to Buscopan. Participants are asked to provide a standardized mood rating at each study visit. In case of a very low rating (1 or 2 on a 10-point scale), further questions will be asked to assess possible suicidal tendencies and ensure the patient’s safety. Several experienced supervisors are available in the background and can be consulted on appropriate measures (e.g., referral to an in-patient clinic) in such cases.

Furthermore, some participants may be on waitlists for psychotherapy elsewhere and participate in the study to bridge the waiting time. Participants who happen to be offered a place to start psychotherapy while completing the trial also discontinue the study treatment.

### Strategies to improve adherence to interventions {11c}

To increase compliance, all participants are given a study visit reminder card at the beginning of treatment. This schedule contains an overview of all planned study visits. Additionally, participants are informed that the full study compensation amount of 100 Euro will only be paid at the last study visit (in all other cases, a partial reimbursement will be paid). Furthermore, we aim to increase treatment adherence in the different treatment arms in several ways; in the Buscopan treatment arms, participants are given the first week’s amount of Buscopan pills at treatment visit 1. At this visit, they are instructed to set a reminder on their smartphone to take the pill each day at the same time. Participants are reminded that pills will be counted after each week of treatment. Furthermore, they are requested to note down if they forgot to take a pill. At the weekly visits, the previous week’s pills will be counted in order to check adherence.

In the emotional writing arms, participants are also told to set reminders for all visits on their smartphones. Participants come to the treatment facility to complete each emotional writing session and meet a trained staff member. This encounter is aimed at instilling a sense of accountability toward the staff and the study process. For the natural control group, short weekly phone calls in which mood ratings are given are further aimed at reducing drop-outs in this group.

### Relevant concomitant care permitted or prohibited during the trial {11d}

Concomitant medication is permitted (as long as the medication is not anti-psychotic or of the benzodiazepine substance group) and was kept constant for 4 weeks prior to study entry and the duration of the study. Concomitant psychotherapy is not permitted. Participants who had psychotherapy in the past need to have terminated therapy at least 4 weeks prior to entry in this study.

### Provisions for post-trial care {30}

After participation in the trial, participants are informed about the option to do an intake-interview for post-trial treatment care at our outpatient treatment center or other suitable facilities. All participants receive a monetary compensation (100 Euro) upon completion.

### Outcomes {12}

Since this trial focusses on major depression, the primary outcome will be depression severity assessed at baseline and post-treatment with the expert rating version of the Montgomery Asberg Depression Scale (MADRS) [50]. Subjective depressive symptoms are assessed with the German version of the Beck Depression Inventory (BDI-II) [51] at baseline, post-intervention, and at follow-up, as a secondary outcome. Using these widely used assessment scales is clinically relevant, as expert ratings and subjective assessments of depressive symptoms can vary significantly. By employing both scales, a more comprehensive understanding of changes in symptomatology can be obtained.

Other clinically relevant self-report outcome measures include the German version of the Pain Disability Inventory (PDI) [52] to measure stress and illness burden; the perceived stress scale (PSS-10) [53], both assessed before the allocation, post-treatment, and at follow-up; and the state-subscale of the State-Trait-Anxiety-Depression-Inventory (STADI) [54], which is assessed at baseline, before the allocation, post-treatment, and at follow-up. Changes in treatment expectations prior to the intervention are measured with the following self-report instruments: Treatment Expectation Questionnaire (TEX-Q) [55] and the Generic rating scale for previous treatment experiences, treatment expectations, and treatment effects (GEEE) [56], both assessed at baseline and after the allocation. Side effects and potential harm is measured before the allocation, post-treatment, and at follow-up with the generic assessment of side-effects scale (GASE) [57] (for a detailed description of all variables, see the “Plans for assessment and collection of outcomes {18a}” section).

### Participant timeline {13}

All four intervention arms, as well as the natural course group, have four assessment points (t0, t1, t6, t7; see Fig. 1). At baseline, all participants give informed consent, undergo diagnostic screening, have a medical examination, and fill in the respective baseline measurements (t0). Randomization (into the congruent, incongruent or natural course group) takes place before the second study visit (t1) at which the four intervention groups receive either a biological or a psychological illness explanation. Subsequently, the assignment to the treatment (pharmacological or psychological) is revealed and a treatment rationale according to random assignment is provided. All subsequent study visits are scheduled. This is followed by four consecutive weeks of treatment in the intervention groups (t2-5). The natural course group is also informed about the randomization at t1, and the short weekly phone calls are scheduled. Once the treatment phase is completed, all participants undergo post-intervention diagnostics and fill in the post-treatment measurements (t6). The procedure is completed by a follow-up assessment 1 week after completion of treatments (t7).

### Sample size {14}

We expect a difference of effects on the primary outcome between treatments with congruent versus incongruent contents of a priori provided treatment rationales. Based on previous research [58], we expect an effect size of Cohen’s *d* > 0.40. Our primary hypothesis comprises of the comparison of the clinical status before and after treatment using the MADRS as the primary outcome. A power of 0.90 and alpha < 0.05 and a 2 × 2 design (treatment rationale, active placebo) requires a minimum of 96 participants. Considering a 20% drop-out/treatment discontinuation, 4 groups of 30 participants are required. The 5th group (natural course) does not serve hypothesis testing. Therefore, this group is not considered in the power calculation. Based on the 5 arms in the study, a total of 150 participants are required.

### Recruitment {15}

Participants will be recruited using flyers in public spaces and healthcare practices, posts in depression self-help forums, social media advertising, and through email lists of the university staff and students. In addition, patients with a primary diagnosis of depression, who are on the waitlist of the university's outpatient clinic, will be approached.

## Assignment of interventions: allocation

### Sequence generation {16a}

Each participant is allocated a number in ascending order from 0 to 149. Participants will be randomized using the Python Package randomization [59] by setting a seed and randomly assigning the 150 subjects to the five treatment arms. A reproducible script is available and will be published after completion of data collection.

### Concealment mechanism {16b}

The allocation (treatment vs. natural course group) as well as the specific allocation within the treatment groups (biological vs. psychological illness rationale; pharmacological vs. psychological placebo treatment) will remain concealed via opaque, sealed envelopes until the start of the treatment phase.

### Implementation {16c}

One investigator, who has no contact with the participants, runs the custom-made script which randomly allocates numbers 0–149 (*N* = 150) to the five conditions. These allocations, including the allocation to the active or control group, the allocation to the treatment rationale (biological or psychological), and the allocation to the treatment condition (pharmacological or psychological placebo), are kept in separate sets of sealed, opaque envelopes. All envelopes are prepared and sealed prior to the start of the study by the investigator.

Once a participant is included in the study, they receive a randomization number (0–149 in ascending order) from the staff member who completed the diagnostic interview. None of the staff, who administer the treatments or assessments, have access to the complete allocation list. The clinician who administers the illness rationale opens the envelope containing the information about whether the participant is allocated to the psychological or biological illness rationale or to the natural course group, before meeting the participant for the second appointment. The envelope indicating the treatment allocation is opened in front of the participant after the illness rationale was given. This ensures that the illness rationale is provided without knowledge of whether the participant receives a congruent or incongruent treatment afterwards.

## Assignment of interventions: blinding

### Who will be blinded {17a}

Participants will be blinded to the congruency condition. Blinding (to group allocation and congruency condition) of the outcome assessors (during baseline and post-assessments) is achieved by having separate clinicians administer the outcome assessments and the illness and treatment rationales. Clinicians who provide the illness rationale will be blinded to the participants’ treatment allocation up to the point at which the respective treatment allocation is revealed. The research staff members who administer the treatment afterwards are blinded to the congruency condition as well.

### Procedure for unblinding if needed {17b}

Unblinding is permissible if a participant becomes a drop-out or shows a marked increase in depressive symptoms and/or suicidal tendencies and is removed from the study. All participants will be debriefed orally and in writing about the aims of the study and group allocation after the study is completed. All participants are also debriefed about the congruency condition and receive both illness rationales upon study completion.

## Data collection and management

### Plans for assessment and collection of outcomes {18a}

#### Primary outcome

The primary outcome is the change in depression severity as measured by the expert rating version of the Montgomery Asberg Depression Scale (MADRS) [50]. The MADRS assesses the following: apparent sadness, reported sadness, inner tension, reduced sleep, reduced appetite, concentration difficulties, lassitude, inability to feel, pessimistic thoughts, and suicidal thoughts. Each item is scored on a scale from 0 (no/hardly any difficulties) to 6 (extreme difficulties). Total scores between 7 and 19 indicate a mild depression, scores between 20 and 34 moderate depression, and a score above 34 severe depression [60]. This scale was shown to achieve high reliability of total and item scores in depressed populations [61]. Blinded and trained clinicians undertake the MADRS assessment at baseline and post-intervention. The outcome assessors are trained to rate the MADRS scores alongside the depression section in the Structured Interview for DSM-5 (SCID-5) diagnoses. A random selection of videotaped MADRS ratings will be rated a second time by independent raters to establish inter-rater reliability.

#### Secondary outcomes

A change from baseline in subjective depressive symptoms is measured using the aggregate score on the German version of the Beck Depression Inventory (BDI-II) [51]. It shows high reliability, with Cronbach’s alphas ranging from 0.84 to 0.90 in German clinical and non-clinical samples [62]. Additionally, the following secondary outcomes will be assessed:

- A change from baseline in subjective disability is measured using the aggregate score of a modified version of the German Pain Disability Inventory (PDI) [52]. This version of the PDI was shown to have high internal consistency (Cronbach’s alpha of 0.93) [52].
- The state-subscale score of the State-Trait-Anxiety-Depression-Inventory (STADI) will be used to assess a change from baseline in anxious-depressive symptoms [54]. The STADI also has high reliability in all subscales [63].
- The aggregate score on the perceived stress scale (PSS-10) is used to assess changes from baseline in subjective stress experience [53]. Cronbach’s *α* was 0.84 in a German sample, indicating good reliability [64].
- The generic assessment of side-effects scale (GASE) will be used to assess experience and attribution of side effects with respect to both treatment types [57]. The GASE was shown to have high internal consistency with Cronbach’s *α* of 0.89.
- Treatment expectations are assessed using the Treatment Expectation Questionnaire (TEX-Q) [55], which shows good internal consistency for all subscales and the sum score (Cronbach’s *α* = 0.71–0.92) [65] and the generic rating scale for previous treatment experiences, treatment expectations, and treatment effects (GEEE) [56]. Psychometric properties of this scale are currently under evaluation.

#### Pre-defined covariates

There are several trait variables, which were shown to influence placebo responses. Some of these variables will be measured and assessed as covariates. They are assessed only at baseline.

- The trait-subscale of the State-Trait-Anxiety-Depression-Inventory (STADI) will be used to control for anxious-depressive symptoms as a stable personality trait [54].
- A German version [66] of the Somatosensory Amplification Scale (SSAS) [67] will be assessed to control for tendencies to experience and amplify somatic and visceral sensations. The original scale showed adequate internal consistency (Cronbach’s *α* = 0.82) [67].
- We will also control for the sensitivity to pleasant incentives using the behavioral inhibition/approach system sensitivity scale (BIS/BAS) [68]. Internal consistency was adequate for the German version [69] with a Cronbach’s *α* of 0.76 (BIS) and 0.79 (BAS) in a German population sample [70].
- Finally, pre-existing experience with psychotherapy and antidepressants, measured with the “prior experience” subscale of the GEEE [56].
- Objective stress will be assessed in two ways: (1) salivary cortisol awakening response and (2) salivary alpha-amylase (sAA) activity, as markers for HPA-axis and sympathetic activity. The cortisol samples will be taken on two consecutive mornings (immediately after waking, 30 and 45 min after awakening) following the baseline assessment.

All other data is collected by the trained research staff. The research staff is blinded at baseline (no allocation has taken place yet). At all other assessment points, the research staff is blinded to the congruency condition. Since all secondary outcomes are self-reported, participants are instructed to fill in the questionnaires in the digital platform (Lime Survey), which makes data transfer from paper–pencil questionnaires into the digital platform obsolete. As part of the diagnostic process at baseline, the BDI-II is also assessed in paper–pencil format, which will later be used to check for consistency between the paper–pencil and digital entries at baseline.

### Plans to promote participant retention and complete follow-up {18b}

The full compensation for study participation and inconveniences (e.g., travel costs) will be paid at the last assessment point (follow-up) to increase motivation to complete all treatment visits. All participants will be equipped with an appointments card and be encouraged to save appointments in their calendars at the beginning of the study in order to promote retention at all assessment points. We also combine several study visits where possible (e.g., pre-treatment measurements and first treatment), in order to reduce the number of study visits to a manageable number, whilst keeping treatment visits close to usual treatment schedules (i.e., one visit per week). Participants who wish to discontinue the study will be asked to fill in a form to assess reasons for discontinuation (e.g., time commitment, adverse effects, other) and will also receive partial compensation. This data will be used to assess whether missing data is likely to be in relation to the interventions themselves.

### Data management {19}

All survey data is entered digitally by the participant via the web-based Lime Survey platform (Lime Survey, Hamburg, Germany) at our study facility. All participant data is anonymized with encryption-based pseudonymization using a software solution (ALIIAS) developed by the TRR/CRC 289, which integrates with the Lime Survey platform and allows for a two-factor authentication via hardware security tokens [71]. The pseudonymization procedure is in accordance with the Declaration of Helsinki, European Directives 2001/20/EC, 95/46/EC, Regulation (CE) No 45/2001 and the General Data Protection Regulation (GDPR, 2016/679) of the EU. The anonymized data is stored centrally on the server of the University Duisburg-Essen. Because the self-report measures are collected in the Lime Survey platform, only valid values can be entered. Nonetheless, range checks of all instruments will be performed as part of the analysis process.

The expert ratings collected during the diagnostic interviews are recorded on paper and entered into a password secured digital document after the appointment. After each diagnostic interview, research staff double check if MADRS scores were calculated correctly by the clinician in the paper version and transferred correctly into the digital document. Again, range checks will be performed before the analysis.

### Confidentiality {27}

Each participant receives a randomization number and a pseudonymized code upon randomization. The randomization number is used to refer to the participant during the trial in our internal procedures. No personal information stored against this number. The pseudonymized code is generated using the ALIIAS anonymization system and all survey data is stored against this code in the Lime Survey cloud [71]. The code can only be traced back to the personal information with a hardware key, which is kept in a locked location that only study staff have access to (for a detailed description of the ALIIAS pseudonymization process, see [71]).

Emails and email addresses of potential participants are kept in our study email account (university email account) and only the research staff in this study, who have signed a confidentiality agreement have access to this account. Handwritten documentation about each participant’s study visits (appointment check lists, diagnostics and other written material) is kept in folders that are locked in a file-drawer in the locked study room, which only the research staff of this study have access to. The data is kept for a maximum of 10 years and is continually kept in a locked location at the study center at the University of Marburg. Personal information (i.e., name, email address, phone number) used to contact participants is kept separately from the written documentation and the pseudonymised data contained in the ALIIAS system and is destroyed after trial. Any documentation that is saved digitally in our local server, for example to track the progress of the trial, does not contain personal information and is password-secured. Participants who wish to be referred to and contacted by the outpatient clinic after the trial give their consent to pass on their contact details to the outpatient clinic.

### Plans for collection, laboratory evaluation, and storage of biological specimens for genetic or molecular analysis in this trial/future use {33}

Cortisol and alpha amylase samples will be collected at our outpatient clinic and sent to the laboratory at the University of Essen for analysis.

## Statistical methods

### Statistical methods for primary and secondary outcomes {20a}

Multivariate procedures with repeated measures will be used to test the main hypothesis (*F*-statistics). The main analysis of interest is the difference in mean change in the primary outcome (MADRS score) from baseline to post-intervention measurement. In addition, linear mixed-effect models with baseline adjustment will be calculated, based on the generalized covariance matrix. Main effects for group, time, and the group-time interaction will be analyzed. The main analyses are based on intention-to-treat (ITT) models. The predefined covariate variables such as subjective pre-existing attitudes of the participants will be included as covariates. Secondary outcomes of interest, namely subjective depressive symptoms, disability, state anxiety, and perceived stress, will be analyzed with the same strategy as described above.

### Interim analyses {21b}

No interim analyses are planned. Data collection will be terminated once the *N* = 150 is reached.

### Methods for additional analyses (e.g., subgroup analyses) {20b}

Several predefined variables (trait anxiety, SSAS, BIS/BAS, GEEE prior experience) will be subjected to subsequent moderator analyses in order to better understand specific mechanisms within the expectation and symptom change process. Equally, certain secondary outcome variables (e.g., state anxiety or perceived disability) and process measures (mood) may mediate change processes and therefore may be subject to additional mediator analyses.

### Methods in analysis to handle protocol non-adherence and any statistical methods to handle missing data {20c}

We will follow the steps outlined by the National Research Council (US) and summarized by Little and colleagues (2012) on the treatment of missing data in clinical trials [72, 73]. In order to keep the integrity of the randomization process and provide a realistic estimate of the treatment effects, we will use ITT and analyze all randomized participants regardless of treatment adherence [74]. Sensitivity analyses will be carried out to assess the impact of missing values in the outcome variable on the results and to decide on a strategy for handling missing data if necessary. If missing data is “missing-at-random” (MAR), multiple imputation is employed to model the missing data in the ITT analysis in order to retain variability in the outcome variables [72, 75–77].

### Plans to give access to the full protocol, participant-level data, and statistical code {31c}

The full protocol and statistical codes are provided on request. Participant-level datasets will be provided to experts on request after thorough clearing to ensure privacy of participants.

## Oversight and monitoring

### Composition of the coordinating center and trial steering committee {5d}

This trial has been approved by the Ethics Committee of the medical chamber of the state of Hessen, Germany (Ethik-Kommission der Landesärztekammer Hessen; reference number: 2019–1349-evBO). The coordinating center for clinical studies at the University of Marburg coordinates this clinical trial locally. The steering committee is comprised of the principal investigators of the Collaborative Research Centre 289-Treatment Expectation (https://treatment-expectation.de/en/projects-people/research-projects; https://gepris.dfg.de/gepris/projekt/422744262).

### Composition of the data monitoring committee, its role and reporting structure {21a}

The data monitoring committee has been established 2021 and consists of external clinician-scientists and psychometricians, all of whom have no affiliation with the trial or the funding body. The committee meets once every 6 months to oversee the procedures and highlight any changes that might need to be made to ensure data quality and participant safety on an ongoing basis.

### Adverse event reporting and harms {22}

No harm is expected from participation in the trial. There are no known side effects for emotional writing. Buscopan can produce some side effects in rare cases (1 in 100 may experience dry mouth, constipation, blurred vision, fast heart rate; very rare: painful red eye and loss of vision, difficulty urinating). Generally, participation in a clinical trial can be distressing and may lead to temporary changes in mood and depressive symptoms. All participants, including control group participants, are contacted weekly to monitor changes in mood and experience of side effects. In case a participant is reporting adverse events, such as a drastic increase in negative mood, suicidal ideation, or other unintended effects, participants will be referred to senior clinicians at the outpatient clinic to ensure safety. Data about adverse events and drop-outs will be reported to the data monitoring committee on an ongoing basis. Side effects will be monitored with the GASE (see above).

### Frequency and plans for auditing trial conduct {23}

External auditing is done by the data monitoring committee on a 6 monthly basis. In terms of internal auditing, weekly meetings with the research staff are held to plan and schedule the workload and discuss possible issues related to the participants progressing through the trial. Monthly graphs depicting participants’ inclusion status and ongoing recruitment strategies are also discussed.

### Plans for communicating important protocol amendments to relevant parties (e.g., trial participants, ethical committees) {25}

Any necessary protocol modifications will be submitted to the overseeing ethics committee for approval. Protocol changes will also be published in the preregistration of this study (registered at www.clinicaltrials.gov; ID: NCT04719663).

### Dissemination plans {31a}

The results of this clinical trial are planned to be published in scientific journals in the field of clinical psychology, psychiatry, and clinical practice. Furthermore, the results will be disseminated to the public through the science communications platforms of the CRC (e.g., website, Twitter, YouTube). Scientific talks and presentations for researchers, healthcare practitioners, and/or the public present another forum for dissemination of the results.

## Discussion

Studies and clinical trials on anti-depressant treatments have shown that placebo arms reveal impressive results rivaling those found in the verum arms [2, 4, 5, 78]. It is yet unknown whether treatment effects can be disentangled from placebo effects and what role common treatment factors, such as pre-treatment expectations, play in producing treatment effects. In this trial, we assess the ways in which clinician-patient communication may help to optimize treatment expectations in the treatment of depression. Specifically, this trial assesses the effects of congruent vs. incongruent illness rationales on treatment effects of two active placebo treatments for major depression with the aim of improving treatment effects.

We hypothesize that illness rationales that are congruent with the subsequent placebo treatment will show larger treatment effects (reduction of depressive symptoms) than illness rationales that are incongruent with the subsequent placebo treatment. Specifically, a psychological causal explanation of depression leads to a better outcome than a biological explanation if a psychological active placebo intervention is provided. Biological causal explanations of depression lead to a better outcome than psychological explanations if an active placebo intervention is provided as treatment. We further hypothesize that the mechanism by which treatment-congruent illness explanations lead to better outcomes is through a modification in the patients’ treatment outcome expectations. Biological rationales increase the expectations for the outcome of pharmacological treatments, while psychological rationales increase positive outcome expectation for psychological interventions. Finally, we hypothesize that inter-individual differences in the effect are associated with pre-treatment experiences and expectations, depression severity, and comorbid anxiety.

Common treatment factors, such as outcome expectations, credibility perceptions, and the therapeutic alliance, have been shown to affect treatment success in pharmacological and psychological trials [28, 30, 31]. We chose to focus on the clinician-patient communication, because the clinician-patient interactions are an integral part of common healthcare procedures and are therefore likely to affect common treatment factors [79, 80]. Yet, little is known about the communicative devices that may work to enhance treatment responses.

Recent experimental research suggests that a therapist’s display of warmth and competence can influence treatment expectations in a therapeutic setting, with participants reporting higher expectations for improvement when therapists display both warmth and competence [81]. These findings highlight the importance of considering therapist behavior and characteristics in shaping patient expectations in therapy.

Furthermore, research on placebo interventions has shown that a convincing treatment rationale which considers patients’ expectations, hopes, and learning history that is delivered by a trustworthy and credible clinician who believes in the remedial qualities of the intervention is able to produce similar treatment effects as other evidence-based interventions [12, 79, 82–85]. We believe a treatment rationale that is in line with the illness model will produce more fruitful grounds for a patient to expect and experience a positive treatment response, irrespective of what the treatment might be (pharmacological or psychological).

Leading up to the current trial, we conducted a pilot study, in which the illness rationales were provided via an animated video. The results showed that both illness explanations (biological vs. psychological causes) were rated similarly in terms of credibility. This is perhaps reflective of the observation that the narrative around illness explanations for depression in the media and in medical practice seems to occur along the biological vs. psychological divide and rarely encompass a bio-psycho-social illness explanation [34]. In contrast to the pilot, in which the credibility of the illness rationale itself was tested, this trial utilizes illness and treatment rationales that are provided face-to-face by clinicians, in order to create a more externally valid context. Moreover, we measure the perceived warmth and competence of clinicians and research staff during key interactions, providing additional insight into the underlying mechanisms of expectation change emerging from the research on common treatment factors.

### Design

Using the BDI-II as a secondary outcome measure for subjective depressive symptoms will further shed light on possible disparities between expert-rated depressive symptoms and the subjective experience of depressive symptoms, which have previously been observed in meta-analytic studies [5]. With other secondary outcomes, such as state anxiety and perceived stress, we are able to assess how expectation modulation and the subsequent interventions aimed at treating depressive symptoms may also interact with related processes (e.g., stress management) and symptoms (e.g., anxiety).

Moderator analyses will explore whether customized approaches in communication could provide an added benefit, for example, for people with high levels of trait anxiety or those with negative previous treatment experiences. Mediator analyses will provide insights into the processes and mechanisms (e.g., expectation updating) by which communication might be able to alter the strength of the placebo response in the treatment of depression.

### Strengths

This trial has several strengths in terms of design and methodology. We intend to investigate and modulate placebo mechanisms directly, using a highly ecological study design with clear implications for clinical work. Few studies in the placebo field account for the natural variation of symptoms [10]. Thus, the inclusion of the natural course control group will allow us to observe whether changes in depressive status could be due to regression to the mean. Based on ethical considerations, this group is kept comparatively small (1/5 of the anticipated sample) in order to give more participants the chance to receive an active placebo treatment. The exclusion criteria are kept to a minimum in order to preserve external validity.

Furthermore, the trial has a 2 × 2 balanced design in the intervention conditions; the chances of being randomized in one of the four treatment arms are equal. This is important as treatment expectations can already be influenced by unequal chances (and thereby unequal prospects for the participant) of being randomized into the different conditions [86].

The blinding procedure presents another strength of this trial, albeit demanding logistically. The separation of outcome raters and clinicians who deliver the rationales for each participant ensures blinding of the outcome (MADRS) raters throughout the trial. Furthermore, clinicians who deliver the illness rationale are blinded to the treatment condition at the time they deliver the illness rationale to the patients. This blinding procedure ensures that there is no knowledge of whether a congruent or incongruent treatment will follow, to avoid bias in the clinician’s delivery of the illness rationale. In addition, the research staff that deliver the interventions after the rationale are different members of staff than the outcome assessors and the clinicians who provide the rationale. This ensures their blinding to the congruency condition, minimizing potential biases in the delivery of the placebo interventions.

### Limitations and future directions

Potential limitations of this trial include the following: the complicated blinding procedure might also present a potential weakness of the trial. In order to preserve blinding of all clinicians and research staff involved, it is necessary to involve different members of staff at nearly every point of interaction with the participant. From a therapeutic relationship-building point of view, it might be more effective if each participant sees only one clinician throughout the whole study [84]. On the other hand, this procedure may not only increase internal validity but also render greater external validity: in general healthcare, it is common for one patient to have several points of contact with care professionals and associated staff, in particular in inpatient settings. For instance, a patient may call a medical secretary to make an appointment, see both a nurse and a doctor during the appointment, and interact with pharmacists who may also offer advice.

Secondly, confirmation bias and factors related to social desirability cannot be completely avoided. Patients in either treatment condition may respond in socially desirable ways, irrespective of whether they received a congruent or incongruent treatment rationale. To counter this, participants are instructed to answer as honestly as possible during the clinician-interactions and the self-report assessments. It is explained that the answers they give have no negative consequences on their participation in the trial. Furthermore, in each assessment session, it is highlighted that the answers on the self-report measures are anonymized, so that it is not possible to track the answers to a specific person. Participants fill in the surveys on their own, in order to avoid feeling supervised, which might increase pressure to give answers perceived as socially desirable. Finally, biological stress markers complement psychological variables, and are less prone to social desirability.

Thirdly, patients`pre-existing preferences in combination with the context (i.e., the research facilities of our outpatient clinic) may have differential effects on the credibility of the two placebo treatments. The context of the outpatient clinic, known as a large psychotherapy provider, may be more in line with the psychological intervention arm. We counteract this potential effect by increasing the emphasis on the medical context within the study. There is an extensive medical examination (duration: 1 h), in a room that contains typical medical items and sensory inputs, that participants would be familiar with from their doctor’s office: medical examination table, various medical examination utensils, smell of disinfectant. To further increase the emphasis on the medical context, contraindications for the pharmacological treatment condition are assessed during this medical interaction. It is further highlighted that the medication used in this study is only available at pharmacies. Future research could further investigate how to harness such context effects to produce stronger placebo responses for either intervention. Patients’ pre-expectations are assessed and controlled in separate analyses.

Finally, it could conceptually be argued that comparing the treatment outcomes of a placebo pill relative to a psychological placebo for the treatment of major depression is akin to comparing apples and oranges, as it is not clear what the effective active ingredients of psychotherapy are or if psychotherapy’s effectiveness comes down to common factors [31]. However, the choice between therapy and medication or a combination of the two is the reality that patients with major depression face. The results of this study will shed light on whether using communication as a tool for modifying treatment expectations is similarly important both in pharmacological and psychological treatments for depression.

## Conclusion

To summarize, the results of this trial will increase knowledge about using communication to modify treatment expectations and improve the effects of pharmacological and psychological placebo interventions in the treatment of depression. Focusing on improving patients’ treatment expectations is important because it is an obligation of the healthcare profession to put the patient in the best possible position to benefit from any given treatment. Ultimately, we hope that the results of this trial will improve clinician-patient interactions in order to produce the best possible outcome for patients suffering from depression.

## Trial status

Recruitment started on April 13, 2021. Recruitment will approximately be completed by November 2023.

### Supplementary Information

**Additional file 1. **

**Additional file 2. **

**Additional file 3. **SPIRIT checklist.

## Data Availability

The project’s investigators and the investigators of the overarching projects of the CRC will have access to the final dataset. The CRC is further committed to sharing best research practices and outcomes with the other project partners (within the CRC) and the wider scientific community as defined in Transparency and Openness Promotion Guidelines (http://cos.io/top; Level 2). Publishing of data (e.g., in PsychData, openneuro.org) and analysis code (e.g., on github.com) will ensure transparency and reproducibility within and across research sites. Of special role will be the support of the Leibniz Institute ZPID and their Open science facilities (Leibniz-Psychology.org) that are particularly developed for psychometric data.

## Abbreviations

BDI-II: Beck Depression Inventory-II
BIS/BAS: Behavioral Inhibition/Behavioral Approach System
CBT: Cognitive behavioral therapy
CRC: Collaborative research center
GASE: Generic Assessment of Side-Effects
GEEE: Generic rating scale for previous treatment experiences, treatment expectations, and treatment effects
ITT: Intention-to-treat
MDD: Major Depressive Disorder
MADRS: Montgomery Asberg Depression Scale
PDI: Pain Disability Index
PSS-10: Perceived Stress Scale-10
SCID-5: Structured Clinical Interview for DSM-5
SSAS: Somatosensory Amplification Scale
STADI: State-Trait-Anxiety-Depression-Inventory
TEX-Q: Treatment Expectation Questionnaire
