# Supplementary material for: The role of the communicated treatment rationale on treatment outcome: study protocol for a randomized controlled trial
Source: Trials. 2023 Aug 17;24:540. doi: 10.1186/s13063-023-07557-w (PMC10433650; doi:10.1186/s13063-023-07557-w)
Supplement: Supplementary file 1 — Additional file 1. [file 13063_2023_7557_MOESM1_ESM.pdf]

Ethik-Kommission bei der Landesärztekammer Hessen

Landesärztekammer Hessen Hanauer Landstraße 152 60314 Frankfurt/Main

Psychotherapie-Ambulanz Marburg
Herrn Prof. Dr. Winfried Rief
Frau Dr. Gudrun Schürmann
Frau Dr. Gesine Rothmund
Gutenbergstraße 18
35032 Marburg

Hanauer Landstraße152 60314 Frankfurt am Main
Postfach 60 05 66 60335 Frankfurt am Main
Telefon: 069 / 97672 – 209
Telefax: 069 / 97672 – 377
E-Mail: ethikkommission@laekh.de
Internet: www.laekh.de

vorab per Cloud

Ihr Zeichen:

(bitte immer angeben)
Unser Zeichen:
V/1/sja
2019-1349-evBO

Datum:
17. April 2020

The modulatory role of communicated treatment rational on treatment expectancy effects in depression

Nachgereichte Unterlagen
Ihr Schreiben vom 2. März 2020, hier eingegangen am 5. März 2020

Sehr geehrter Herr Professor Rief,
sehr geehrte Frau Dr. Schürmann,
sehr geehrte Frau Dr. Rothmund,

wir bestätigen den Eingang des oben aufgeführten Schreibens vom 2. März 2020. Damit sind die Forderungen der Ethik-Kommission aus dem Schreiben vom 25. September 2019 angemessen umgesetzt worden.

Gegen die Durchführung der Studie

> **The modulatory role of communicated treatment rational on treatment expectancy effects in depression**

bestehen nunmehr weder berufsethische noch berufsrechtliche Bedenken soweit die untenstehenden Hinweise noch Berücksichtigung finden.

Es wird darauf hingewiesen, dass datenschutzrechtliche Aspekte von Forschungsvorhaben durch die Ethik-Kommission grundsätzlich nur kursorisch geprüft werden und dieses Schreiben nicht die Konsultation des zuständigen behördlichen Datenschutzbeauftragten ersetzt.

Es wird davon ausgegangen, dass die Datensicherheitsmaßnahmen gewährleistet, die datenschutzrechtlichen Vorschriften - insbesondere § 27 BDSG und § 24 HDISG einschließlich der Bereithaltung eines Datenschutzkonzeptes – beachtet und die nach EU-DSGVO sowie HDISG bzw. BDSG erforderlichen technischen und organisatorischen Maßnahmen eingehalten werden.

...

Die Ethik-Kommission bittet um zeitnahe Unterrichtung über alle schwerwiegenden oder unerwarteten unerwünschten Ereignisse, die während der Studie auftreten und die Sicherheit der Studienteilnehmer oder die Durchführung der Studie beeinträchtigen könnten. Dies gilt auch, wenn die Studie aus unvorhergesehenen Gründen abgebrochen wird.

Es wird darauf hingewiesen, dass Änderungen oder Erweiterungen des Versuchsplanes der Ethik-Kommission anzuzeigen sind und gegebenenfalls eine erneute Beratung erforderlich wird. In den einzureichenden Studienunterlagen sind die Änderungen und/oder Erweiterungen deutlich zu kennzeichnen; es müssen alle Änderungen nachvollziehbar aus den Unterlagen hervorgehen (insbesondere Streichungen).

Die Ethik-Kommission bittet außerdem nach Abschluss des Forschungsvorhabens um einen Bericht mit der Mitteilung der bei der Studie gewonnenen Ergebnisse.

Die ärztliche und juristische Verantwortung des Studienleiters und der an der Prüfung teilnehmenden Ärzte bleibt entsprechend der Beratungsfunktion der Ethik-Kommission durch unsere Stellungnahme unberührt.

Der Beratung lagen die in der Anlage aufgeführten Unterlagen zugrunde, es haben die in der Anlage aufgeführten Mitglieder der Ethik-Kommission mitgewirkt.

Mit freundlichen Grüßen
i.A.

Prof. Dr. med. S. Harder
Vorsitzender der Ethik-Kommission

Hinweise

I.

Die Unterlagen der regulären Probanden-und Unfallversicherung, die über die HDI Global SE abgeschlossen werden soll, sind nach Erhalt unverzüglich vorzulegen.

Das Anmeldeformular, welches vom KKS Marburg eingereicht wird, liegt entgegen Ihrer Ankündigung nicht den Unterlagen bei und ist zeitnah nachzureichen.

II.

Die Ethik-Kommission erwartet, dass der nachfolgende Hinweis noch Berücksichtigung findet. Einer erneuten Vorlage der entsprechend geänderten Unterlage zur Freigabe seitens der Ethik-Kommission bedarf es nicht.

Auf Seite 6 der Probandeninformation muss „Der Zweck und die Rechtsgrundlage der Datenverarbeitung ergibt sich aus der wissenschaftlichen Fragestellung zur Rolle von Krankheitsinformationen für den Behandlungsverlauf." ergänzt / korrigiert werden. Die Rechtsgrundlage der Datenverarbeitung ist die Einwilligung des Studienteilnehmers.

...

Anlage

Zur Beratung vorgelegte Unterlagen

Dokument	Version	Datum
Prüfplan		12.08.2019<br>05.03.2020
Antrag an die Ethikkommission der Landesärztekammer Hessen		12.08.2019<br>05.03.2020
Probandeninformation zur Studie		05.08.2019<br>05.03.2020
Einwilligungserklärung		09.09.2019<br>05.03.2020

Ihr Schreiben vom 12. August 2019, hier eingegangen am 19. August 2019
Ihr Schreiben vom 4. September 2019, hier eingegangen am 4. September 2019
Ihr Schreiben vom 2. März 2020, hier eingegangen am 5. März 2020

An der Beratung mitwirkende Mitglieder

Prof. Dr. med. Sebastian Harder, Vorsitzender (Klinischer Pharmakologe)
PD Dr. med. Horst Baas (Neurologe)
Prof. Dr. med. Werner Rettwitz-Volk (Kinder- und Jugendmediziner)
PD Dr. med. Jochen Graff (Klinischer Pharmakologe)
Prof. Dr. med. Elke Jäger (Internistin)
Prof. Dr. med. Michael Weber (Innere Medizin/Kardiologie)
Dr. Hanns Ackermann (Dipl. Math.)
Dr. iur. Annkatrin Helberg-Lubinski (Rechtsanwältin)
Dr. rer. med. Jennifer Engler, MPH
Herr Prof. Dr. med. Dr. iur. Reinhard Dettmeyer (Rechtsmediziner)

Die Ethik-Kommission bei der Landesärztekammer Hessen verarbeitet Ihre personenbezogenen Daten im Einklang mit den jeweils anzuwendenden gesetzlichen Datenschutzanforderungen. Die Informationspflichten nach Art. 13 und Art. 14 DSGVO im Rahmen unserer Tätigkeit haben wir für Sie auf unserer Homepage unter https://www.laekh.de/images/Aerzte/Rund_ums_Recht/Ethikkommission/DSGVO_Informationspflichten_Ethik_Kommission.pdf zusammengefasst.
