# Supplementary material for: The role of the communicated treatment rationale on treatment outcome: study protocol for a randomized controlled trial
Source: Trials. 2023 Aug 17;24:540. doi: 10.1186/s13063-023-07557-w (PMC10433650; doi:10.1186/s13063-023-07557-w)
Supplement: Supplementary file 2 — Additional file 2. [file 13063_2023_7557_MOESM2_ESM.docx]

**English translation of the ethical approval document:**

V/1/sja

**2019-1349-evBO**

**Date:** 17. April 2020

**The modulatory role of communicated treatment rationale on treatment expectancy**

effects in depression

Supplementary documents

Your letter of March 2, 2020, received here on March 5, 2020.

Dear Professor Rief,

Dear Dr. Schürmann,

Dear Dr. Rothmund,

We confirm the receipt of the above-mentioned letter dated March 2, 2020. Thus, the demands of the Ethics Committee from the letter dated September 25, 2019 have been appropriately implemented.

There are no longer any professional ethical or professional legal concerns regarding the implementation of the study "The modulatory role of communicated treatment rational on treatment expectancy effects in depression," provided that the information below is still taken into account.

It should be noted that data protection aspects of research projects are generally only superficially examined by the Ethics Committee and this letter does not replace consultation with the responsible regulatory data protection officer.

It is assumed that the data security measures are ensured, the data protection regulations - in particular § 27 BDSG and § 24 HDISG including the provision of a data protection concept - are observed and the necessary technical and organizational measures required by EUDSGVO as well as HDISG or BDSG are adhered to.

The Ethics Committee requests prompt notification of all serious or unexpected adverse events that occur during the study and that could affect the safety of study participants or the conduct of the study. This also applies if the study is unexpectedly terminated.

Please note that changes or expansions to the study plan must be reported to the Ethics Committee and may require further consultation. The changes and/or expansions must be clearly indicated in the submitted study documents; all changes must be traceable from the documents (especially deletions).

Furthermore, upon completion of the research project, the Ethics Committee requests a report with the communication of the study's findings.

The medical and legal responsibility of the study director and the participating physicians remains unaffected by our opinion in accordance with the advisory function of the Ethics Committee.

The consultation was based on the documents listed in the attachment, and the members of the Ethics Committee listed in the attachment contributed.

Best regards,

On behalf of

Prof. Dr. med. S. Harder

Chairman of the Ethics Committee.

Notes:

I.

The documents for the regular participant and accident insurance, which is to be taken out

through HDI Global SE, must be submitted promptly upon receipt.

Contrary to your announcement, the registration form, which will be submitted by KKS Marburg,

is not included in the documents and must be submitted promptly.

II.

The Ethics Committee expects that the following note will still be taken into account. It is not

necessary to resubmit the correspondingly amended document for approval by the Ethics

Committee.

On page 6 of the participant information, "The purpose and legal basis of the data processing

result from the scientific question about the role of disease information for the course of

treatment" must be added/corrected. The legal basis for data processing is the consent of the

study participant.

Attachment:

Documents submitted for consultation

Assessment plan 12.08.2019; 05.03.2020

Application to the Ethics Committee of the State Medical Association Hessen 12.08.2019; 05.03.2020

Participant information for the study 05.08.2019; 05.03.2020

Informed consent 09.09.2019; 05.03.2020

Your letter of August 12, 2019, received here on August 19, 2019

Your letter of September 4, 2019, received here on September 4, 2019

Your letter of March 2, 2020, received here on March 5, 2020

Members of the Ethics Committee who participated in the consultation:

Prof. Dr. med. Sebastian Harder, Chairman (Clinical Pharmacologist)

PD Dr. med. Horst Baas (Neurologist)

Prof. Dr. med. Werner Rettwitz-Volk (Pediatrician and Adolescent Medicine Specialist)

PD Dr. med. Jochen Graff (Clinical Pharmacologist)

Prof. Dr. med. Elke Jäger (Internal Medicine Specialist)

Prof. Dr. med. Michael Weber (Internal Medicine/Cardiology)

Dr. Hanns Ackermann (Dipl. Math.)

Dr. iur. Annkatrin Helberg-Lubinski (Lawyer)

Dr. rer. med. Jennifer Engler, MPH

Prof. Dr. med. Dr. iur. Reinhard Dettmeyer (Forensic Pathologist)

The Ethics Committee at the State Medical Association of Hessen processes your personal data in accordance with the applicable legal data protection requirements. We have summarized the information obligations under Art. 13 and Art. 14 of the GDPR in connection with our activities for you on our homepage at the following link:

https://www.laekh.de/images/Aerzte/Rund_ums_Recht/Ethikkommission/DSGVO_lnformationspflichten_Ethik_Kommission.pdf
